# B Cell-Based Vaccine Transduced With ESAT6-Expressing Vaccinia Virus and Presenting α-Galactosylceramide Is a Novel Vaccine Candidate Against ESAT6-Expressing Mycobacterial Diseases

**DOI:** 10.3389/fimmu.2019.02542

**Published:** 2019-10-29

**Authors:** Bo-Eun Kwon, Jae-Hee Ahn, Eun-Kyoung Park, Hyunjin Jeong, Hyo-Ji Lee, Yu-Jin Jung, Sung Jae Shin, Hye-Sook Jeong, Jung Sik Yoo, EunKyoung Shin, Sang-Gu Yeo, Sun-Young Chang, Hyun-Jeong Ko

**Affiliations:** ^1^Laboratory of Microbiology and Immunology, College of Pharmacy, Kangwon National University, Chuncheon, South Korea; ^2^Department of Biological Sciences, Kangwon National University, Chuncheon, South Korea; ^3^Department of Microbiology, Institute for Immunology and Immunological Disease, Brain Korea 21 PLUS Project for Medical Science, Yonsei University College of Medicine, Seoul, South Korea; ^4^Division of Vaccine Research, Center for Infectious Disease Research, Korea National Institute of Health (KNIH), Korea Centers for Disease Control and Prevention (KCDC), Cheongju, South Korea; ^5^Sejong Institute of Health and Environment, Sejong, South Korea; ^6^Laboratory of Microbiology, College of Pharmacy and Research Institute of Pharmaceutical Science and Technology (RIPST), Ajou University, Suwon, South Korea

**Keywords:** *Mycobacterium kansasii*, *Mycobacterium tuberculosis*, non-tuberculous mycobacteria, ESAT6, vaccine, α-galactosylceramide

## Abstract

Early secretory antigenic target-6 (ESAT6) is a potent immunogenic antigen expressed in *Mycobacterium tuberculosis* as well as in some non-tuberculous mycobacteria (NTM), such as *M. kansasii*. *M. kansasii* is one of the most clinically relevant species of NTM that causes mycobacterial lung disease, which is clinically indistinguishable from tuberculosis. In the current study, we designed a novel cell-based vaccine using B cells that were transduced with vaccinia virus expressing ESAT6 (vacESAT6), and presenting α-galactosylceramide (αGC), a ligand of invariant NKT cells. We found that B cells loaded with αGC had increased levels of CD80 and CD86 after *in vitro* stimulation with NKT cells. Immunization of mice with B/αGC/vacESAT6 induced CD4^+^ T cells producing TNF-α and IFN-γ in response to heat-killed *M. tuberculosis*. Immunization of mice with B/αGC/vacESAT6 ameliorated severe lung inflammation caused by *M. kansasii* infection. We also confirmed that immunization with B/αGC/vacESAT6 reduced *M. kansasii* bacterial burden in the lungs. In addition, therapeutic administration of B/αGC/vacESAT6 increased IFN-γ^+^ CD4^+^ T cells and inhibited the progression of lung pathology caused by *M. kansasii* infection. Thus, B/αGC/vacESAT6 could be a potent vaccine candidate for the prevention and treatment of ESAT6-expressing mycobacterial infection caused by *M. kansasii*.

## Introduction

Non-tuberculous mycobacteria (NTM) are one of the mycobacteria species which cause pulmonary disease as a common manifestation (1). *Mycobacterium kansasii* belongs to NTM species and is one of the major causative agent of NTM lung disease (2). Symptoms of *M. kansasii* are mild under single infection, but it is known that more severe symptoms occur when contracted along with other illnesses such as inflammatory pseudotumor (3), sarcoidosis (4), and HIV (5). Especially, it has been reported that in Brazil, most patients who acquire lung disease caused by NTM had previously received tuberculosis treatment (6). These reports implied that NTM was closely associated with other diseases, and therefore is one of the important factors in pulmonary infection.

Bacillus Calmette-Guerin (BCG) is the only approved live attenuated vaccine strain induced from *M. bovis* through multiple sub-culturing for a long period of time (7). The protective efficacy of BCG against tuberculous meningitis and tuberculosis (TB) is well-known in children, however, protection for primary infection or latent infection in adults seems poor (8). Also, BCG vaccination did not provide protection against NTM infection (9). Due to this limitation of BCG, more persistent research is needed to identify novel vaccine candidates.

Early secretory antigenic target-6 (ESAT6) is a protein encoded by a gene located in the region of difference 1, which is expressed in *M. tuberculosis* but not in BCG (10). ESAT6 has sufficient immunogenicity in both humans and mice post *M. tuberculosis* infection (11). Interestingly, some NTM species, including *M. kansasii* also contain genes for ESAT6 homolog. In the present study, we expressed ESAT6 in B cells using ESAT6-expressing vaccinia virus to deliver ESAT6 antigen to B cells, and presented α-galactosylceramide (αGC), an invariant natural killer cell (iNKT) ligand, on CD1d molecule of B cells. Previous studies have suggested that a B cell vaccine which expressed tumor antigen showed potent anti-tumor effect facilitated by activated NKT cells (12, 13).

In the current study, we developed an ESAT6-expressing B cell-based vaccine which was loaded with αGC (B/αGC/vacESAT6) and assessed its preventive and therapeutic effect in a murine model of *M. kansasii* infection.

## Materials and Methods

### Construction of Vaccinia Virus Vector Expressing ESAT6

*ESAT6* gene of *M. tuberculosis* strain H37Rv with human optimized codon was synthesized and cloned into vaccinia virus delivery vector PVVT1-C7L (PVVT1-C7L-Tpa-esat6) which contains *tPA* gene for secretion of intracellular signal peptide. Sfi1 restriction enzyme was used for cloning. PVVT1-C7L-Tpa-esat6 was transformed to *E. coli* DH5 competent cells for amplification. The expression of *ESAT6* gene was confirmed by PCR using the following primers; 5′-TTT GAA GCA TTG GAA GCA ACT-3′ (VVTK-F) and 5′-ACGTTGAAATGTCCCATCGACT-3′ (VVTK-R).

### Preparation of Recombinant Vaccinia Virus Expressing ESAT6

**Figure 1:**
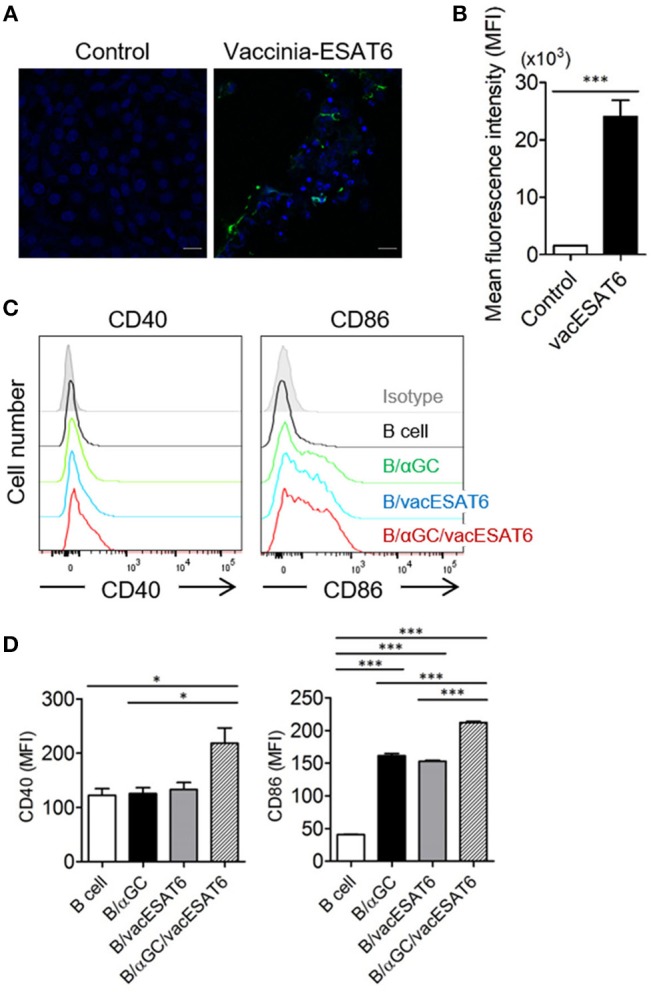
B/αGC/vacESAT6 up-regulates co-stimulatory molecules on B cells. **(A,B)** Vero cells were transduced with vaccinia-ESAT6 (vacESAT6) at a multiplicity of infection (MOI) of 1. Transduced cells were fluorescently stained for ESAT6 (green) and counterstained with DAPI (blue) for nuclei which were analyzed by confocal microscopy to detect the expression of ESAT6 (scale bar = 20 μm). **(A)** Representative confocal images and **(B)** Representation of the evaluation of green fluorescent area. **(C,D)** B220^+^ cells were isolated from splenocytes of naïve C57BL/6 mice. Isolated B220^+^ cells were transduced with vacESAT6 at a MOI of 1 and/or loaded with 1 μg/ml of αGC and then co-cultured with naïve splenocytes for 24 h. Incubated cells were stained to examine the expression of B220, CD40, and CD86. Expression levels of CD40 and CD86 in B220^+^ cells were analyzed by flow cytometry. **(C)** Representative flow cytometry histogram and **(D)** summary of mean fluorescence intensity of CD40 and CD86 expression in B cells. ANOVA. *p < 0.05, ***p < 0.001.

Vero cells in 12-well plates were infected with vaccinia virus (KCCM11574P) at a multiplicity of infection (MOI) of 0.02 for 2 h, and the infected Vero cells were transfected with PVVT1-C7L-Tpa-esat6 plasmid using Lipofectamine 2000 (Thermo Fisher Scientific, Waltham, MA, USA) transfection reagent for 4 h. Vero cells were incubated for 3–4 days to observe the cytopathic effects, and recombinant viruses were obtained by plaque isolation. For high efficacy and purity, recombinant vaccinia virus expressing ESAT6 (vacESAT6) was concentrated by ultracentrifugation. The expression of ESAT6 protein by Vero cells and isolated B cells after transduction with vacESAT6 was confirmed by confocal microscopy (Figures 1A,B, Supplementary Figure 1).

### Preparation of B Cell-Based Vaccine and Immunization of BCG

B220^+^ cells were magnetically purified from splenocytes of naïve C57BL/6 mice using CD45R/B220 biotin (BD biosciences, California, USA) and anti-biotin microbeads (Miltenyi Biotec, Bergisch Gladbach, Germany) according to the manufacturer's instructions. Labeled cells were purified through LS column (Miltenyi Biotec, Bergisch Gladbach, Germany). Isolated B220^+^ cells (2 × 10^7^ cells seeded) were transduced with vacESAT6 (MOI of 1) for 2 h and then loaded with αGC (Enzo life sciences, New York, USA) (1 μg/ml) for 22 h and incubated in a CO_2_ incubator. After washing three times with PBS, mice were immunized with cultured cells (B cell-based vaccine) by tail vein injection. As for the comparison group, mice were intramuscularly immunized with BCG (10^5^ CFU/mouse).

To confirm preventive effect of Bvac, mice were either immunized with BCG by intramuscular injection at a 10^5^ CFU/mouse or administered with Bvac by tail vein injection at day 0 for the priming and day 7 for the boost. At day 14, mice were challenged with 10^7^ CFU/mouse of *M. kansasii*. To confirm the therapeutic effect of Bvac, mice were infected with 10^7^ CFU of *M. kansasii* per mouse at Day 0, and were administrated with BCG or Bvac at 3 days post-infection. The mice were boosted with Bvac at 7 days post-infection. We analyzed histology, protein levels and bacterial loads from lung and liver after 14 days following *M. kansasii* infection.

### Murine Infection Model of *M. kansasii*

C57BL/6 mice were purchased at 6–7 weeks of age from Charles River Laboratories (Orient Bio Inc., Seongnam, Korea). All animal experiments, including the *M. kansasii* challenge experiment, were approved by the Institutional Animal Care and Use Committee of Kangwon National University (Permit Number: KW-160201-4). A hypervirulent *M. kansasii* SM#1 clinical isolate was used for challenge *in vivo* (14). To induce infection, mice were intravenously injected with *M. kansasii* (10^7^ CFU/mouse). We checked the bodyweight and survival rate of mice every day following *M. kansasii* infection.

The lungs and liver of infected mice were isolated for determining the bacterial count in these organs at 2 weeks post *M. kansasii* infection. Lungs were homogenized in 1X PBS containing 0.04% tween 80 and liver was homogenized in 1X PBS containing 1 mM EDTA (125 mg of tissues/ml). The homogenized supernatants were 10-fold serial diluted in Difco™ Middlebrook 7H9 Broth (BD biosciences, California, USA) containing ADC [Sodium Chloride (Duchefa, BH Haarlem, The Nederlands), Dextrose (SHOWA, Gyoda, Japan), Bovine Albumin Fraction V (MPBio, Santa Ana, USA), Catalase (Sigma-Aldrich, St. Louis, USA)] and each diluent was drop cultured in Difco™ Middlebrook 7H10 Agar (BD biosciences, California, USA) containing OADC [Sodium Chloride, Dextrose, Bovine Albumin Fraction V, Catalase, Oleic acid (Sigma-Aldrich, St. Louis, USA)]. Smear plates were cultured in a 37°C incubator and colonies were counted after 2–3 weeks.

### Isolation of Cells and Measurement of Co-stimulatory Molecules in B Cells

For the isolation of T cells, splenocytes were labeled with CD8α-PE and anti-PE microbeads (Miltenyi Biotec, Bergisch Gladbach, Germany) according to the manufacturer's instructions. The labeled cells were then purified through LS column (Miltenyi Biotec, Bergisch Gladbach, Germany). CD4^+^ T cells were isolated by using the mouse CD4^+^ T cell isolation kit (Miltenyi Biotec, Bergisch Gladbach, Germany). CD11c^+^ DCs were purified by using CD11c^+^ microbeads (Miltenyi Biotec, Bergisch Gladbach, Germany). B220^+^ cells were purified by using CD45R/B220 biotin (BD biosciences, California, USA) and anti-Biotin microbeads (Miltenyi Biotec, Bergisch Gladbach, Germany) from splenocytes of naïve C57BL/6 mice. Purified B cells were transduced with vacESAT6 at a MOI of 1 and/or loaded with 1 μg/ml of αGC and then co-cultured with naïve splenocytes for 24 h. Incubated cells were stained to examine the expression of B220, CD40, and CD86 using antibodies such as APC-conjugated anti-B220 (BD biosciences, California, USA), PE-conjugated isotype control (eBioscience, San Diego, USA), anti-CD40 (Biolegend, San Diego, USA), and anti-CD86 Ab (BD biosciences, California, USA). Cells were analyzed by flow cytometry.

### Measurement of Intracellular Cytokines in CD4^+^ T Cells

For measurement of intracellular cytokines, dendritic cells and CD4^+^ T cells were co-cultured and stimulated for 3 days with heat-killed H37Rv at 0.1 MOI or overnight with anti-CD3 and anti-CD28 antibody in culture media. H37Rv strain was generously provided by Sang-Nae Cho (Yonsei University). Brefeldin A Solution (1000 x) Thermo Fisher Scientific, Waltham, MA, USA) was added for 4 h before harvest and then harvested cells were stained with PerCP-Cy™5.5 rat anti-mouse CD4 (BD biosciences, California, USA) or PE rat anti-mouse CD8α (BD biosciences, California, USA). Stained cells were permeabilized with IC Fixation Buffer (Thermo Fisher Scientific, Waltham, MA, USA) according to the manufacturer's recommendations. Next, permeabilized cells were stained with TNF-α mAb, APC (Thermo Fisher Scientific, Waltham, MA, USA) and IFN-γ mAb (XMG1.2) PE (Thermo Fisher Scientific, Waltham, MA, USA).

The supernatants of homogenized tissues were analyzed for cytokine production using BD™ Cytometric Bead Array (CBA) Mouse Inflammation kit (BD biosciences, California, USA) according to the manufacturer's instructions.

### Western Blotting

Total protein lysates of *M. kansasii* were sonicated with PRO-PREP™ protein extraction solution (iNtRON Biotechnology, Daejeon, Korea). Lysates were boiled at 100°C and proteins were separated by performing SDS-PAGE. Proteins were transferred onto PVDF membranes (Millipore, Burlington, USA) and then blocked with 5% skim milk in TBS with tween 20. Next, the membranes were incubated with anti-ESAT6 primary antibody [11G4] (Abcam, Cambridge, USA) and proteins were detected using HRP conjugated goat anti-mouse polyclonal antibody (Enzo life sciences, New York, USA). Membranes were developed using femtoLUCENT™ PLUS-HRP chemiluminescence detection system (G-Biosciences, St. Louis, USA).

### Flow Cytometry

Cells were collected from spleen and stained with markers such as APC-conjugated anti-B220 (BD biosciences, California, USA) and anti-Ly6C (BD biosciences, California, USA) Ab, FITC-conjugated anti-CD11b Ab (BD biosciences, California, USA), PE-conjugated isotype control (eBioscience, San Diego, USA), anti-CD40 Ab (Biolegend, San Diego, USA), and anti-CD86 (BD biosciences, California, USA) Ab. Flow cytometry was performed on a FACSVerse instrument (BD biosciences, California, USA) and data were analyzed using FlowJo software (Flowjo, San Carlos, USA).

### Histology

Mice were sacrificed and lungs were isolated from each group. They were fixed with 4% formalin overnight. Lung tissues were processed using a tissue processor (Leica, Wetzlar, Germany) and then embedded in paraffin. Paraffin-embedded tissue blocks were cut into 5 μm thick sections and stained with hematoxylin and eosin.

### Confocal Microscopy

Uninfected or vaccinia virus-infected Vero cells and B cells were cultured in a 37°C incubator for 24 h. Cells were fixed with 4% paraformaldehyde and stained with anti-ESAT6 Ab (Abcam, Cambridge, USA), and further stained with anti-mouse IgG (H+L), F(ab')_2_ Fragment (Alexa Fluor® 488-conjugated) (Cell signaling, Danvers, USA) or DyLight™ 405 affinipure donkey anti-mouse IgG (H+L) (DyLight™ 405-conjugated). Cells were visualized by confocal microscopy (Carl Zeiss, LSM880 with Airyscan, Zena, Germany).

### Statistics

Statistical analysis was conducted with GraphPad Prism 5.0 (GraphPad Software, La Jolla, USA). Differences between groups were assessed by the Student's *t*-test. Comparisons between multiple-groups were carried out by one-way ANOVA analysis of variance followed by the Bonferroni's multiple comparison test. *P* values < 0.05 were considered as significant at a 95% confidence interval for all analyses.

## Results

### B/αGC/vacESAT6 Upregulated the Expression of Co-stimulatory Molecules on B Cells

It has been previously reported that αGC-loaded B cell-based vaccines expressing tumor antigens showed significant antitumor effects *in vivo* (15, 16). Thus, we decided to adopt this vaccine strategy for the development of preventive and therapeutic anti-mycobacterial vaccine. B cells were transduced with recombinant vaccinia virus expressing ESAT6 (vacESAT6), and the transduced B cells were loaded with αGC. We found that B cells loaded with αGC/vacESAT6 (B/αGC/vacESAT6) (Bvac) increased the expression of co-stimulatory molecules including CD40 and CD86 when they were co-cultured with splenocytes from naïve C57BL/6 mice for 24 h (Figures 1C,D). B cells loaded with αGC or B cells transduced with vacESAT6 increased the expression of CD86, which further increased in B/αGC/vacESAT6 (Figures 1C,D). B cells transduced with vacGFP control virus also increased the expression of CD86 (Supplementary Figure 2) suggesting that infection of vaccinia virus alone could be stimulatory for B cells. These results suggested that activated NKT cells by αGC on B cells as well as vaccinia virus infection activated the B cells to increase the expression of co-stimulatory molecules including CD40 and CD86, which help B cells to function as professional antigen presenting cells to induce effective T cells.

### B/αGC/vacESAT6 Induced CD4^+^ T Cell Responses Against H37Rv

**Figure 2:**
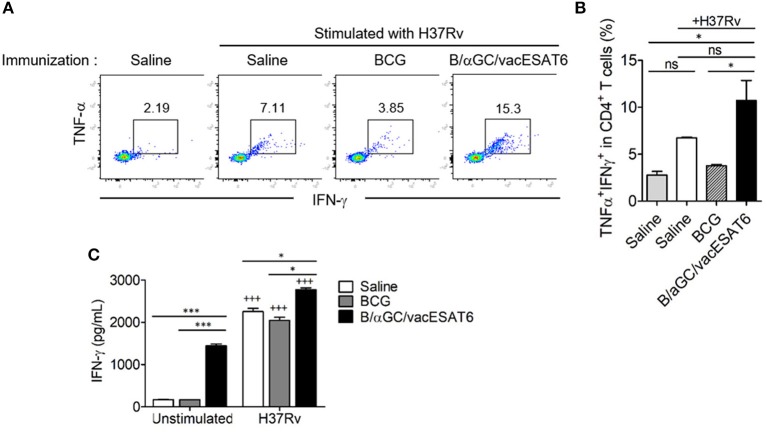
B/αGC/vacESAT6 induced *M. tuberculosis* specific CD4^+^ T cell response. Splenic CD4^+^ T cells were isolated from mice which were immunized with BCG (10^5^ CFU/mouse) or with B/αGC/vacESAT6 (Bvac), and they were co-cultured with CD11c^+^ dendritic cells which were stimulated with heat-killed *M. tuberculosis* H37Rv at 0.1 MOI. After 72 h co-culture, CD4^+^ T cells were analyzed by flow cytometry to detect intracellular TNF-α and IFN-γ production. **(A)** Representative intracellular staining of TNF-α and IFN-γ. **(B)** Summary of TNF-α and IFN-γ secreting CD4^+^ T cells. **(C)** Secreted IFN-γ levels were measured in culture supernatant by ELISA. ANOVA. *p < 0.05, ***p < 0.001, ^+++^*p* < 0.001 compared to unstimulated counterpart.

We next determined whether B/αGC/vacESAT6 induced CD4^+^ T cell response *in vivo*. Groups of mice were immunized with either saline, BCG or B/αGC/vacESAT6, and mice were sacrificed to obtain splenocytes. We analyzed TNF-α- and IFN-γ-producing CD4^+^ T cells after 3 days of co-culturing the splenocytes with dendritic cells pulsed with heat-killed H37Rv. As a result, we found that the percentage of CD4^+^ T cells producing TNF-α^+^ and IFN-γ^+^ was higher in mice vaccinated with B/αGC/vacESAT6 as compared to control and BCG-immunized mice (Figures 2A,B). In addition, IFN-γ production was also increased in the culture supernatant of splenocytes from B/αGC/vacESAT6 group compared to control and BCG group (Figure 2C). These results show that B/αGC/vacESAT6 induced the H37Rv-specific CD4^+^ T cell-mediated cellular immunity which might be critical for the regulation of mycobacterial infection.

### Mice Infected With *M. kansasii* Showed Severe Lung Inflammation

**Figure 3:**
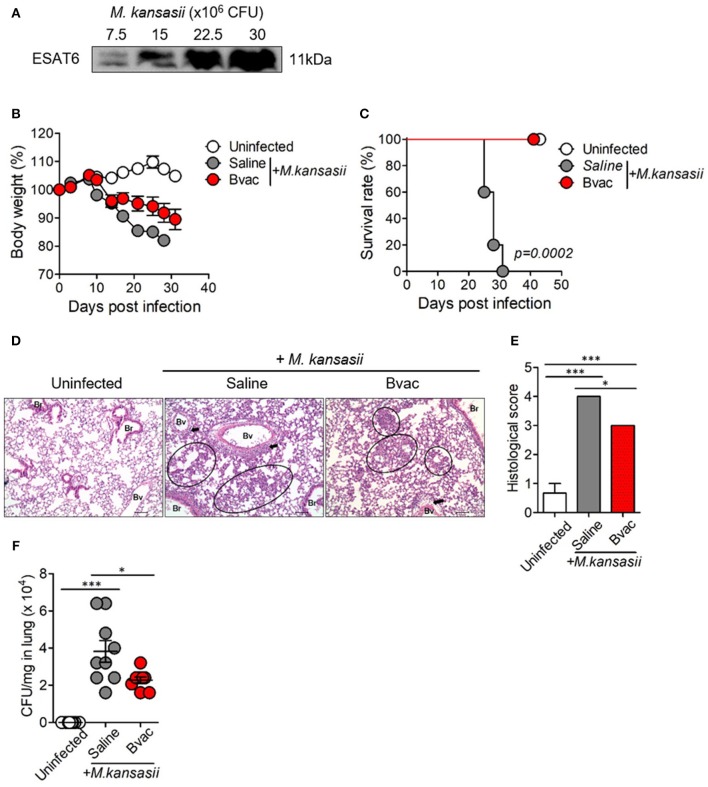
Immunization of mice with Bvac prevented *M. kansasii* infection. **(A)** Expression of 11 kDa ESAT6 obtained from lysates of various CFU of *M. kansasii* by western blotting. **(B–F)** Mice were intravenously immunized with Bvac via tail vein. After 14 days of immunization mice were challenged with *M. kansasii* (10^7^ CFU/mouse) (*n* = 5 for uninfected, *n* = 6 for saline and Bvac group). After 14 days following *M. kansasii* infection, histology and bacterial CFU were determined from lungs. **(B)** Bodyweight. ANOVA. *p < 0.05, *M. kansasii* vs. Bvac+ *M. kansasii*. **(C)** Survival rate. Log-rank test. **(D)** Representative hematoxylin and eosin staining of lung sections from each group of mice (scale bar = 100 μm). Bv (Blood vessel), Br (Bronchus), circle indicates interstitial necrotizing inflammatory foci, and arrow is perivascular inflammatory cell infiltration. **(E)** Histological scores of the lung sections. **(F)** CFU of *M. kansasii* from lung homogenates. ANOVA. *p < 0.05, ***p < 0.001.

*M. kansasii* is one of the NTM which expresses ESAT6 homolog as a major antigen. We confirmed the expression of ESAT6 in *M. kansasii* by western blotting analysis (Figure 3A). We found that the bodyweight of mice significantly decreased with intravenous (i.v) injection of *M. kansasii* (10^7^ CFU/mouse). For humane reasons, mice were monitored two times every day and sacrificed when they weighed <80% of their initial bodyweight. In addition, there was significant lung injury including infiltration of immune cells around bronchial tubes as well as formation of granuloma-like lesions. We also confirmed the presence of bacteria in lungs of *M. kansasii*-infected mice at 2 weeks after infection. On the contrary, immunization of mice with Bvac ameliorated loss of bodyweight and increased survival rate following *M. kansasii* infection (Figures 3B,C). In addition, lungs of mice immunized with Bvac had moderate injury with reduced cell infiltration as compared with non-vaccinated mice after *M. kansasii* infection (Figures 3D,E). Bvac also decreased the bacterial burden in the lungs of *M. kansasii*-infected mice (Figure 3F). We also analyzed the proportion of NK cells and NKT cells in splenocytes by flow cytometry. We confirmed that Bvac immunization increased the percentage of NKT cells as compared with that of non-vaccinated mice as assessed after *M. kansasii* infection (Supplementary Figure 3). We also compared the therapeutic effects of Bvac and BCG vaccine in *M. kansasii*-infected mice. As a result, mice therapeutically treated with BCG and Bvac showed moderate levels of inflammation with reduced cell infiltration and decreased the bacterial burden in the lungs of *M. kansasii*-infected mice (Supplementary Figures 4A,B). Intriguingly, however, immunization of mice with Bvac significantly decreased the bacterial burden in the liver than BCG immunization after *M. kansasii* infection (Supplementary Figures 4C,D). Collectively, we established a murine model of infection of *M. kansasii* and showed that Bvac had preventive effect against *M. kansasii* expressing ESAT6.

### B/αGC/vacESAT6 Had Therapeutic Effects Against *M. kansasii* Infection

**Figure 4:**
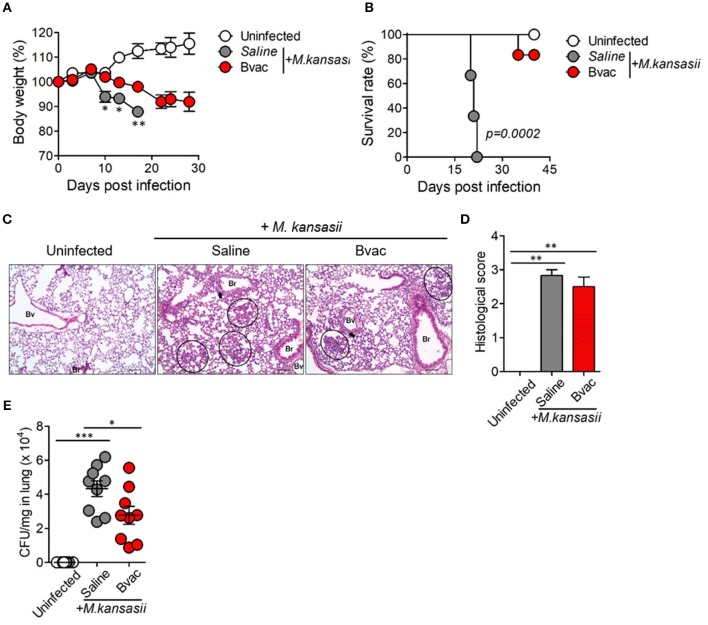
Bvac has therapeutic effect on mice infected with *M. kansasii*. To evaluate therapeutic efficacy of Bvac, mice were infected with 10^7^ CFU of *M. kansasii* per mouse, and Bvac was administered via tail vein injection at day 3 and 7 post infection (*n* = 5 for uninfected, *n* = 6 for saline and Bvac group). After 14 days following *M. kansasii* infection, histology and bacterial CFU were determined from lung. **(A)** Body weights of mice are shown, ANOVA. *p < 0.05, **p < 0.01, *M. kansasii* vs. Bvac + *M. kansasii*. **(B)** Survival rate. Log-rank test. **(C)** Representative hematoxylin and eosin staining of lung sections from each group of mice (scale bar = 100 μm). Bv (Blood vessel), Br (Bronchus), circle indicates interstitial necrotizing inflammatory foci, and arrow is perivascular inflammatory cell infiltration. **(D)** Histological scores of the lung sections. **(E)** CFU of *M. kansasii* from lung homogenates. ANOVA. *p < 0.05, ***p < 0.001.

**Figure 5:**
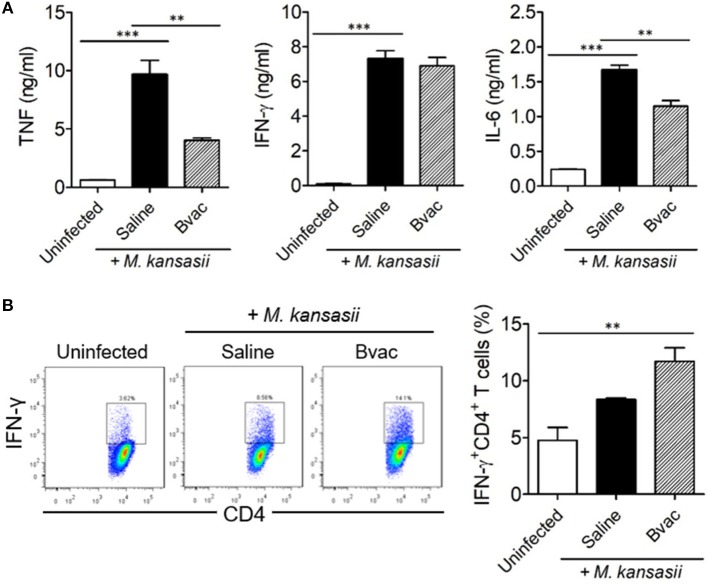
Therapeutic administration of Bvac increased the production of IFN-γ in CD4^+^ T cells in *M. kansasii*-infected mice. Mice were infected with 10^7^ CFU/mouse of *M. kansasii*, and then they were intravenously injected with Bvac at day 3 and 7 post infection. After 14 days following *M. kansasii* infection, lung and spleen were isolated to analyze cytokine production. **(A)** The levels of TNF, IFN-γ, and IL-6 from lung homogenates. **(B)** Splenic CD4^+^ T cells were analyzed for IFN-γ production after *in vitro* stimulation with plate-coated anti-CD3 and anti-CD28 antibodies. Representative histogram (left) and summary (right) for the percentages of IFN-γ producing CD4^+^ T cells. ANOVA. **p < 0.01, ***p < 0.001.

We speculated whether B/αGC/vacESAT6 (Bvac) had therapeutic effects against mice infected with *M. kansasii*. To evaluate therapeutic efficacy of Bvac, mice were i.v. challenged with 10^7^ CFU/mouse of *M. kansasii*, and 14 days later, they were injected with Bvac at day 3 and 7 post-infection. When we checked the bodyweight, mice administered with Bvac showed alleviation in loss of body weight as compared to mice infected with *M. kansasii* (Figure 4A). Further, survival rate increased in mice treated with Bvac (Figure 4B). Histological analysis of lungs of infected mice confirmed the therapeutic effects of Bvac against *M. kansasii* infection (Figures 4C,D). Bacterial load in the lungs was significantly reduced in mice administered with Bvac as compared to *M. kansasii* infected mice (Figure 4E). Also, administered with BCG did not show the therapeutic effect, while Bvac administration reduced bacterial loads in lungs of the infected mice (Supplementary Figure 5). Furthermore, production of TNF and IL-6, which were increased in lungs of infected mice following *M. kansasii* infection, were significantly decreased when administered with Bvac (Figure 5A). Collectively these results suggested that Bvac had a therapeutic effect in mice infected with *M. kansasii*.

### Bvac Increased the Production of IFN-γ in CD4^+^ T Cells in a Therapeutic Mouse Model

Finally, we assessed the production of intracellular cytokines in CD4^+^ T cells after therapeutic treatment with Bvac in *M. kansasii* infected mice. IFN-γ-producing T cells are known to play a key role in resistance against various pathogens including *M. tuberculosis* (17). Mice were infected with *M. kansasii* (10^7^ CFU/mouse), and i.v. injected with Bvac at day 3 and 7 post infection. After 14 days following *M. kansasii* infection, lung homogenates were analyzed for the production of TNF, IFN-γ, and IL-6. Interestingly, although the levels of TNF, IFN-γ and IL-6, were highly increased in mice infected with *M. kansasii*, the levels of TNF and IL-6 were significantly reduced. When CD4^+^ T cells obtained from the spleen of the treated mice were analyzed for IFN-γ production, we found that splenocytes of mice administered with Bvac showed increased production of IFN-γ in CD4^+^ T cells compared to *M. kansasii-*infected mice (Figure 5B). Consequently, these results suggest that Bvac could induce IFN-γ-producing CD4^+^ T cell response, resulting in preventive and therapeutic effects against *M. kansasii* expressing ESAT6 (Supplementary Figure 6).

## Discussion

Tuberculosis is the most dangerous and incurable disease in the world (18–20). Although most patients with *M. tuberculosis* infection can be cured with appropriate treatment with anti-tuberculosis drugs such as isoniazid, rifampin, pyrazinamide, and ethambutol, it is difficult to treat multidrug resistant tuberculosis (MDR-TB) and extensively drug-resistant tuberculosis (XDR-TB) (21). Novel drugs including bedaquiline and delamanid have been introduced to deal with MDR-TB (22). In addition, drug repositioning approaches have provided linezolid, imatinib, and metformin for the treatment of TB patients (23–27). However, since treatment of MDR-TB requires at least 4 months, studies on the development of new anti-tuberculosis drugs are still needed. In this study, we suggest a novel approach to treat mycobacterial infection including *M. tuberculosis* using a therapeutic vaccine.

Currently, the only licensed vaccine for TB is BCG. BCG is made by attenuating *M. bovis*. However, there are several limitations of BCG as TB vaccine since it cannot prevent the development of primary infection and reactivation of latent pulmonary infection. Besides, the efficacy of BCG vaccine has a broad range and limited efficacy in adults. Due to the limitation of BCG vaccine, there is an immediate need for further research to develop a novel mycobacterial vaccine.

NTM is another contagious disease-causing pathogen in humans (17). It has been reported that the incidence of multiple NTM infections and NTM-associated mortality rates have dramatically increased in recent times (28, 29). Non-tuberculous mycobacterial pulmonary disease (NTM-PD) is one of the main conditions caused by NTM (30, 31) and the radiological manifestation of NTM-PD is classified as fibrocavitary form (similar to pulmonary tuberculosis) and nodular bronchiectatic form (similar to MAC pulmonary disease) (32, 33). It has been reported that NTM-PD infection increases with age (34), co-infection with chronic obstructive pulmonary disease (COPD) and asthma, in patients (35).

*M. kansasii* is known as one of the main pathogens causing NTM-PD (36). It has been reported that *M. kansasii*-infected patients are mostly infected with other disorders such as tuberculosis, other types of NTM, and HIV, which are resulting in exacerbated symptoms and weakened immune system (2). Treatment of *M. kansasii* infection is typically by administering rifampin, but sometimes fails due to resistance to rifampin. Ethambutol and isoniazid are also used, but drug resistance against these drugs have also been reported (37, 38). Since NTM-PD is accompanied by other diseases, we presumed that a novel approach using an immunotherapeutic agent or vaccine could be used to treat NTM infection as well as *M. tuberculosis*, which essentially increases host immunity.

To control *M. tuberculosis* or NTM infection, it has been recognized that Th1 response is important. IFN-γ, which is mainly expressed by Th1, supports to activate macrophages and empowers it to successfully degrade invaded bacteria. Additionally, activation of Th1 cells helps B cells to produce antibodies which suppress free bacteria by inducing the formation of immune complexes. However, a recent study reported anomalies of CD4^+^ T cell physiology in NTM-infected host. NTM infected patients, especially when infected with *M. intracellulare* or *M. avium*, showed reduced CD4^+^ T cells in PBMC (39). In addition, the importance of IFN-γ production seems controversial in a mouse infection model. A systemic infection model induced by intravenous injection of *M. kansasii* showed CD4^+^ T cell-dependent reduction of mycobacterial burden in multiple organs. However, intranasal infection revealed no significant alteration of severity between WT and IL-12p40-, CD4-, or IFN-γ- deficient mice, triggered by *M. kansasii* infection (40, 41). These data suggested the restricted contribution of CD4+ T cell function in suppressing *M. kansasii* in intranasally-induced lung infection model. Collectively, these reports imply that the generation of IFN-γ producing CD4^+^ T cell response is crucial to control systemic *M. kansasii* infection similar to other species of mycobacterium.

Recently, vaccines using antigen-presenting cells including dendritic cells and B cells have been suggested to induce strong T cell-mediated immunity (11, 15). B cell vaccine is one of the cell-based vaccine approaches developed by using pathogen-specific antigens to induce diverse immune responses including Th1, cytotoxic T cell, and pathogenic antigen-specific antibody response. To augment CD4^+^ T cell response, αGC, a ligand of NKT cell receptor, was used to load into the CD1d molecules on B cell surface (11).

In the current study, we designed a B cell-based vaccine (B/αGC/vacESAT6), which was transduced by vaccinia virus expressing ESAT6 and loaded with αGC. ESAT6 is a 6 kDa secretory protein and is one of the critical antigens, widely used as a candidate antigen for the development of new TB vaccine. ESAT6 has been shown to have sufficient immunogenicity such as CD4^+^ T cell and CTL responses in both rodents and humans (42). Although ESAT6 is one of the promising antigen candidates, it might inhibit innate immunity by TLR2 binding, and can also inhibit the function of MHC molecules by the phagosomal rupture. However, we could not find any significant adverse effect after the administration of B/αGC/vacESAT6.

The ESAT6 of *M. bovis* and *M. tuberculosis* is identical (43, 44) and the amino acid sequence of ESAT6 homolog of *M. kansasii* is highly similar to that of *M. bovis*. Thus, we presumed that ESAT6 of *M. tuberculosis* could protect *M. kansasii* infection. In the current study, we confirmed that immunization with B/αGC/vacESAT6 ameliorated pulmonary inflammation caused by *M. kansasii* infection. Especially, therapeutic treatment of B/αGC/vacESAT6 decreased bodyweight loss and bacterial load in the lungs following *M. kansasii* infection, as well as increased the survival rate of *M. kansasii*-infected mice. The therapeutic administration of B/αGC/vacESAT6 increased IFN-γ production by CD4^+^ T cells. In addition, B/αGC/vacESAT6 altered the composition of other immune cells in lungs such as CD8 T cells and myeloid cells.

Collectively, we developed a αGC-loaded, ESAT6 expressing B-cell based vaccine (B/αGC/vacESAT6) and confirmed the preventive and therapeutic effect of B/αGC/vacESAT6 vaccine in a murine model of *M. kansasii* infection.

## Data Availability Statement

All datasets generated for this study are included in the article/Supplementary Material.

## Ethics Statement

The animal study was reviewed and approved by Institutional Animal Care and Use Committee of Kangwon National University, Kangwon National University, Chuncheon Gangwon-do 24341, South Korea (Permit Number: KW-160201-4).

## Author Contributions

B-EK and H-JK designed this study. H-SJ, JY, ES, S-GY, SS, H-JL, and Y-JJ contributed with materials and analysis tools. B-EK performed and analyzed *in vivo* animal experiments together with E-KP and HJ. B-EK and J-HA performed data interpretation and discussion. B-EK, J-HA, S-YC, and H-JK wrote the manuscript. All authors reviewed the manuscript.

### Conflict of Interest

The authors declare that the research was conducted in the absence of any commercial or financial relationships that could be construed as a potential conflict of interest.
