# Supplementary material for: B Cell-Based Vaccine Transduced With ESAT6-Expressing Vaccinia Virus and Presenting α-Galactosylceramide Is a Novel Vaccine Candidate Against ESAT6-Expressing Mycobacterial Diseases
Source: Front Immunol. 2019 Oct 29;10:2542. doi: 10.3389/fimmu.2019.02542 (PMC6830241; doi:10.3389/fimmu.2019.02542)
Supplement: Supplementary file 1 [file Presentation_1.PPTX]

## Slide 1
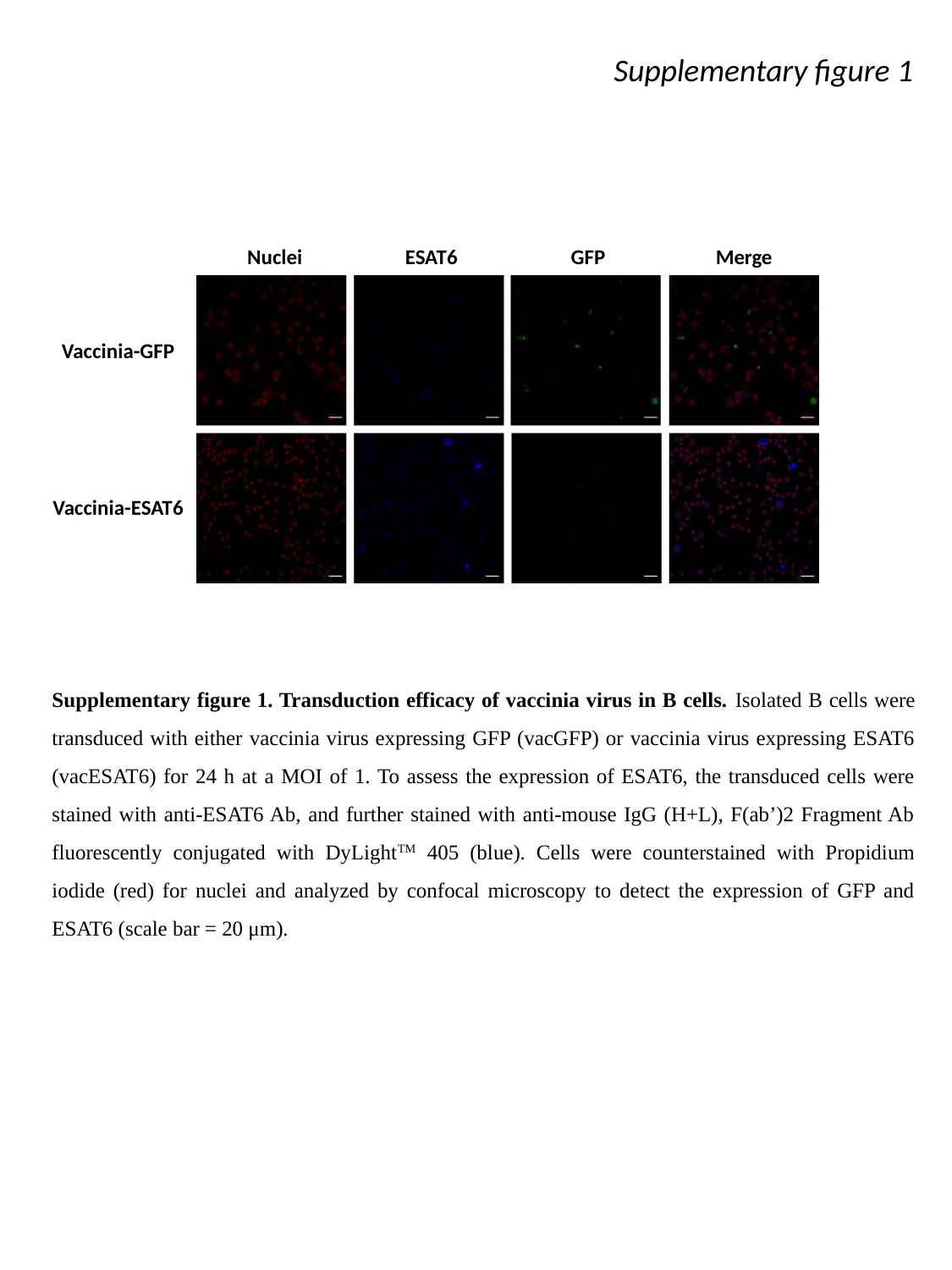

Supplementary figure 1
GFP
Merge
Nuclei
ESAT6
Vaccinia-GFP
Vaccinia-ESAT6
Supplementary figure 1. Transduction efficacy of vaccinia virus in B cells. Isolated B cells were transduced with either vaccinia virus expressing GFP (vacGFP) or vaccinia virus expressing ESAT6 (vacESAT6) for 24 h at a MOI of 1. To assess the expression of ESAT6, the transduced cells were stained with anti-ESAT6 Ab, and further stained with anti-mouse IgG (H+L), F(ab’)2 Fragment Ab fluorescently conjugated with DyLightTM 405 (blue). Cells were counterstained with Propidium iodide (red) for nuclei and analyzed by confocal microscopy to detect the expression of GFP and ESAT6 (scale bar = 20 μm).

## Slide 2
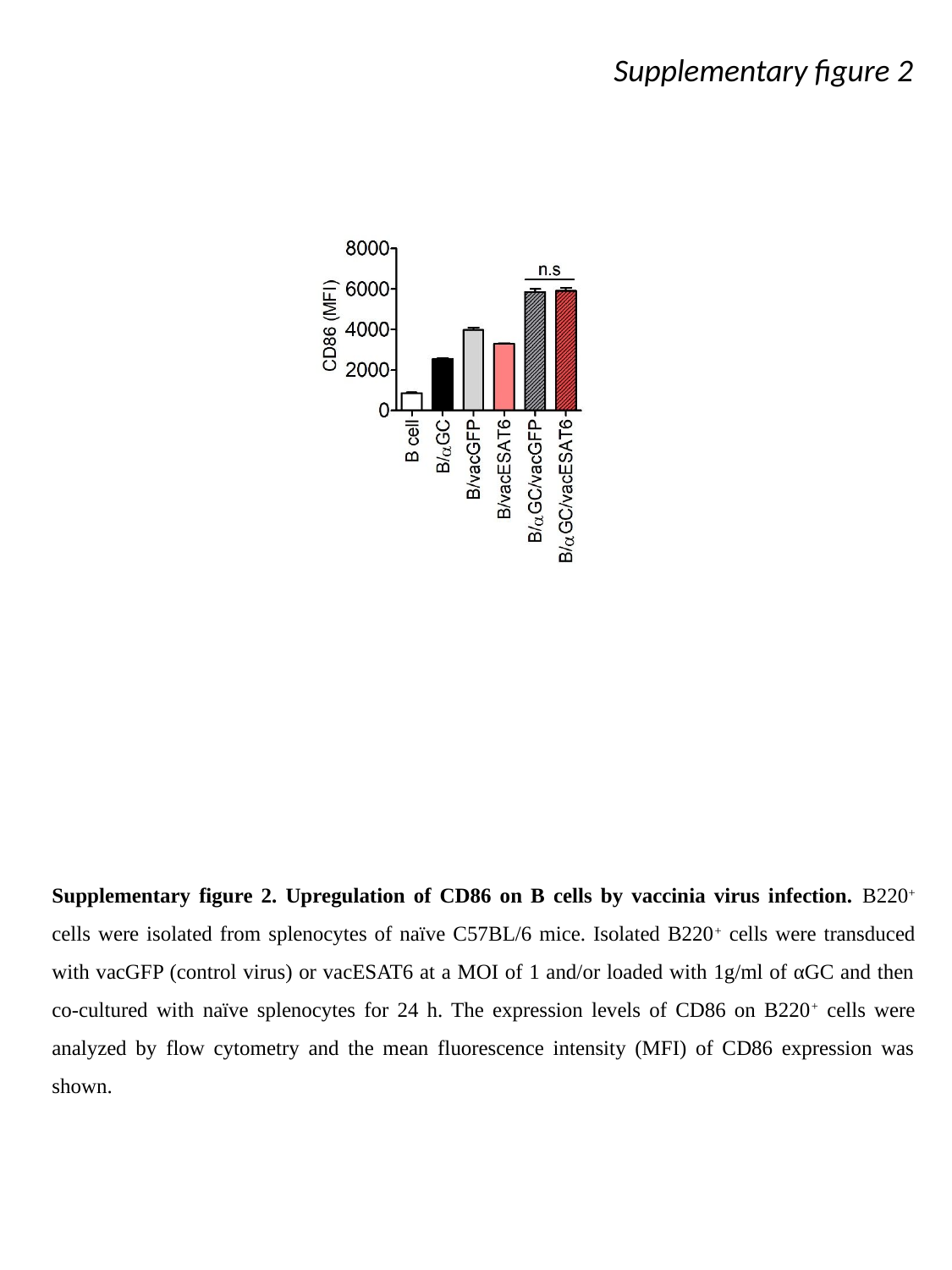

Supplementary figure 2

## Slide 3
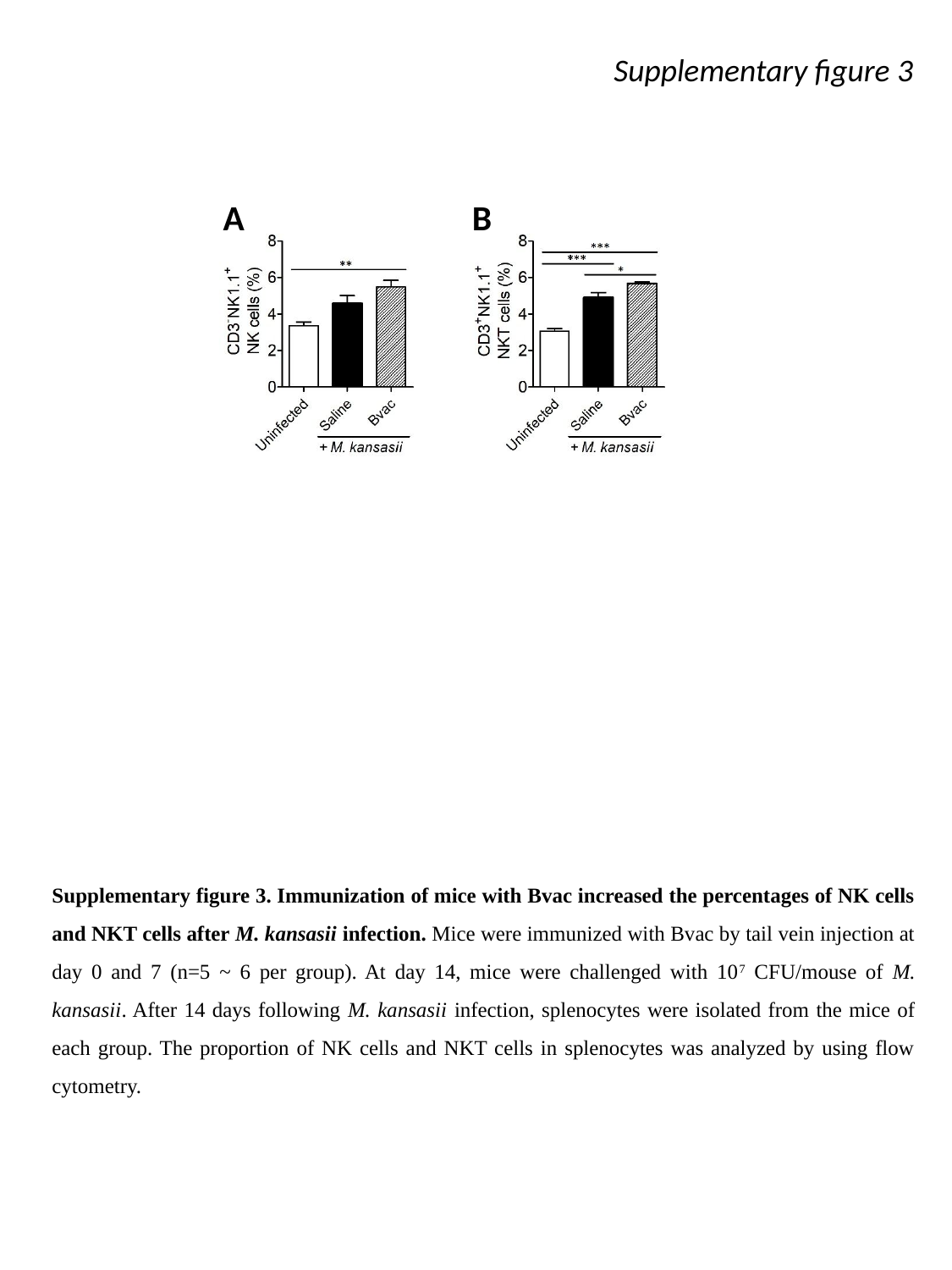

Supplementary figure 3
A
B
Supplementary figure 3. Immunization of mice with Bvac increased the percentages of NK cells and NKT cells after M. kansasii infection. Mice were immunized with Bvac by tail vein injection at day 0 and 7 (n=5 ~ 6 per group). At day 14, mice were challenged with 107 CFU/mouse of M. kansasii. After 14 days following M. kansasii infection, splenocytes were isolated from the mice of each group. The proportion of NK cells and NKT cells in splenocytes was analyzed by using flow cytometry.

## Slide 4
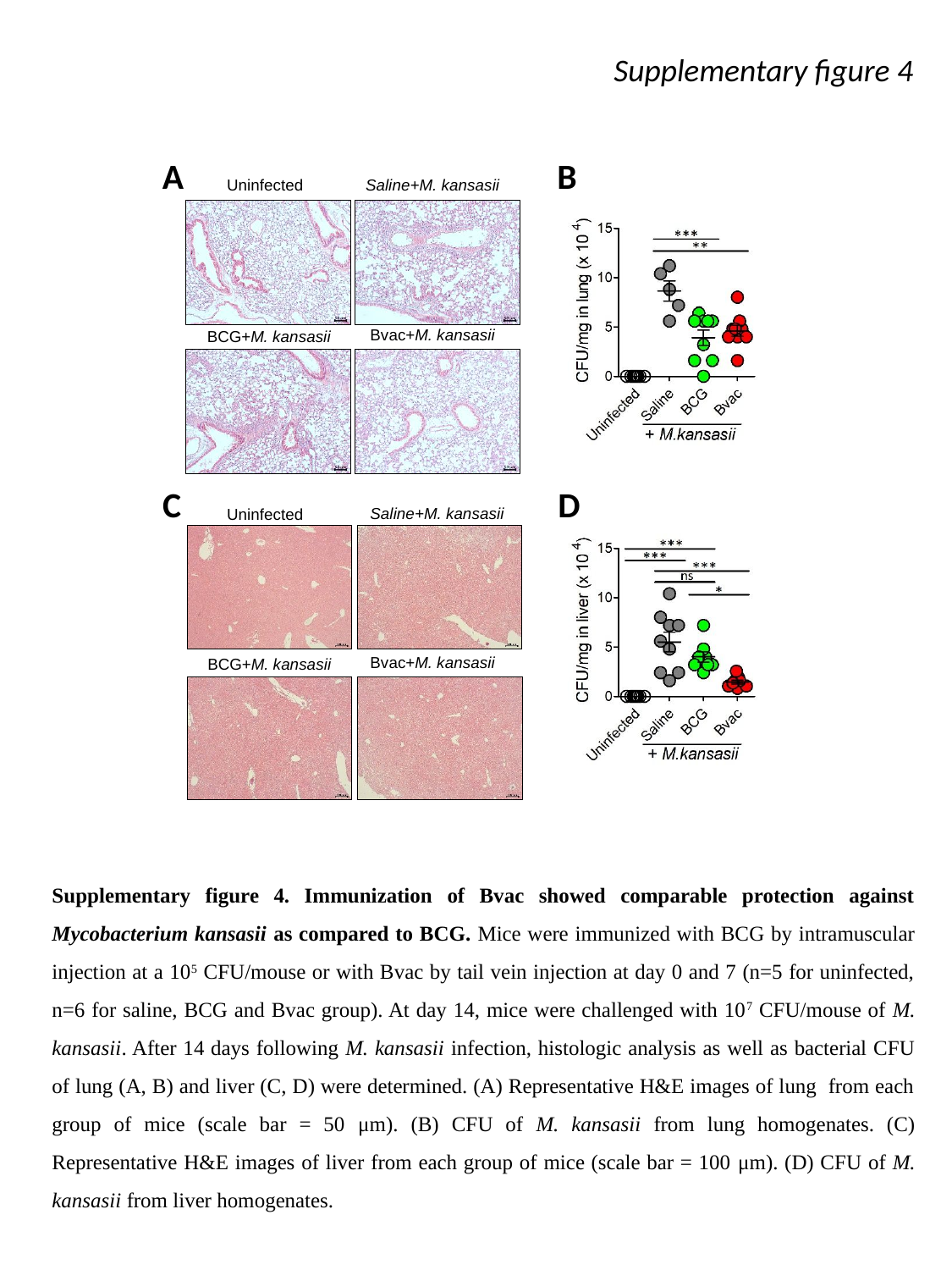

Supplementary figure 4
A
B
Uninfected
Saline+M. kansasii
Bvac+M. kansasii
BCG+M. kansasii
C
D
Saline+M. kansasii
Uninfected
Bvac+M. kansasii
BCG+M. kansasii
Supplementary figure 4. Immunization of Bvac showed comparable protection against Mycobacterium kansasii as compared to BCG. Mice were immunized with BCG by intramuscular injection at a 105 CFU/mouse or with Bvac by tail vein injection at day 0 and 7 (n=5 for uninfected, n=6 for saline, BCG and Bvac group). At day 14, mice were challenged with 107 CFU/mouse of M. kansasii. After 14 days following M. kansasii infection, histologic analysis as well as bacterial CFU of lung (A, B) and liver (C, D) were determined. (A) Representative H&E images of lung from each group of mice (scale bar = 50 μm). (B) CFU of M. kansasii from lung homogenates. (C) Representative H&E images of liver from each group of mice (scale bar = 100 μm). (D) CFU of M. kansasii from liver homogenates.

## Slide 5
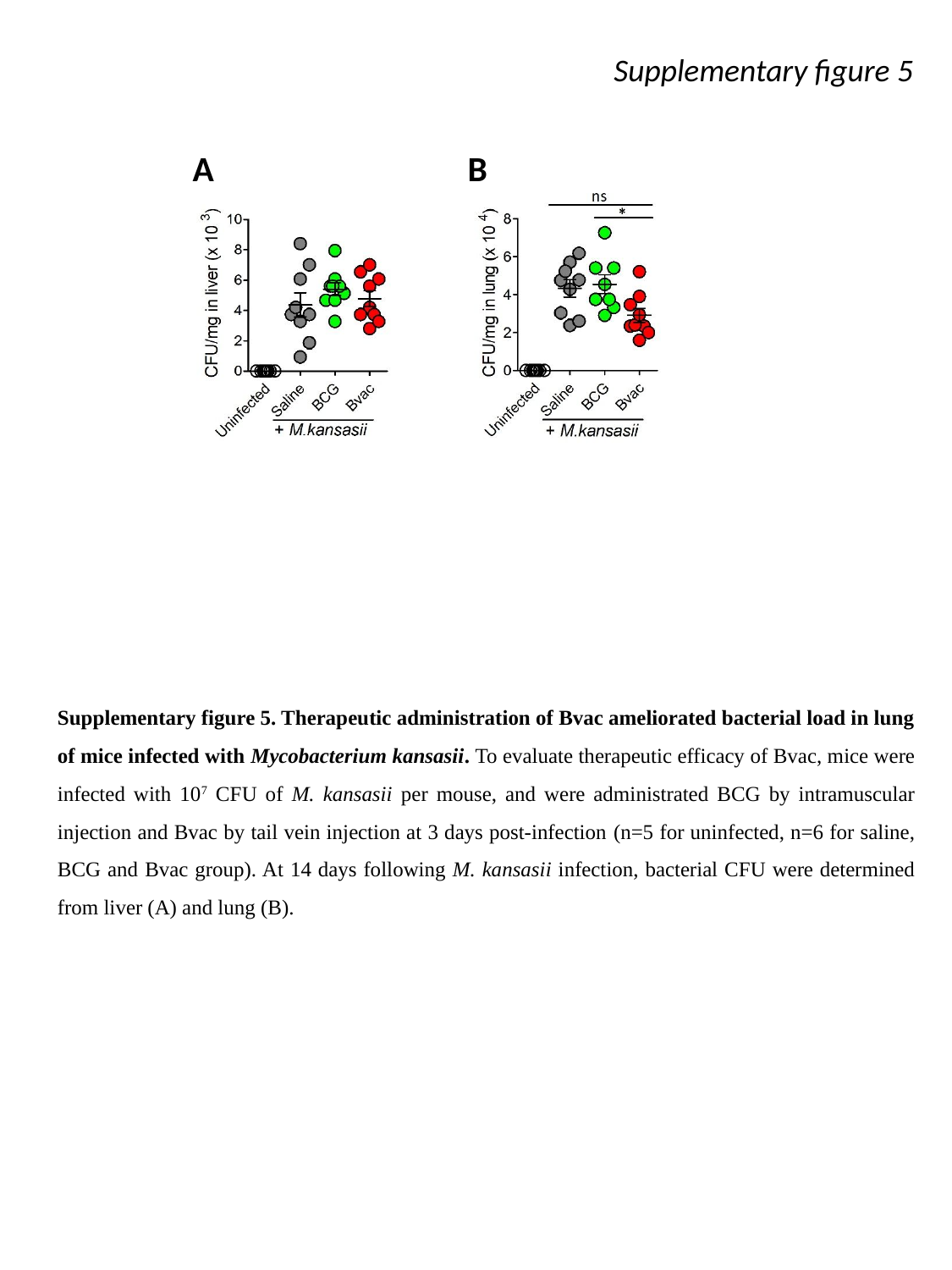

Supplementary figure 5
A
B
Supplementary figure 5. Therapeutic administration of Bvac ameliorated bacterial load in lung of mice infected with Mycobacterium kansasii. To evaluate therapeutic efficacy of Bvac, mice were infected with 107 CFU of M. kansasii per mouse, and were administrated BCG by intramuscular injection and Bvac by tail vein injection at 3 days post-infection (n=5 for uninfected, n=6 for saline, BCG and Bvac group). At 14 days following M. kansasii infection, bacterial CFU were determined from liver (A) and lung (B).

## Slide 6
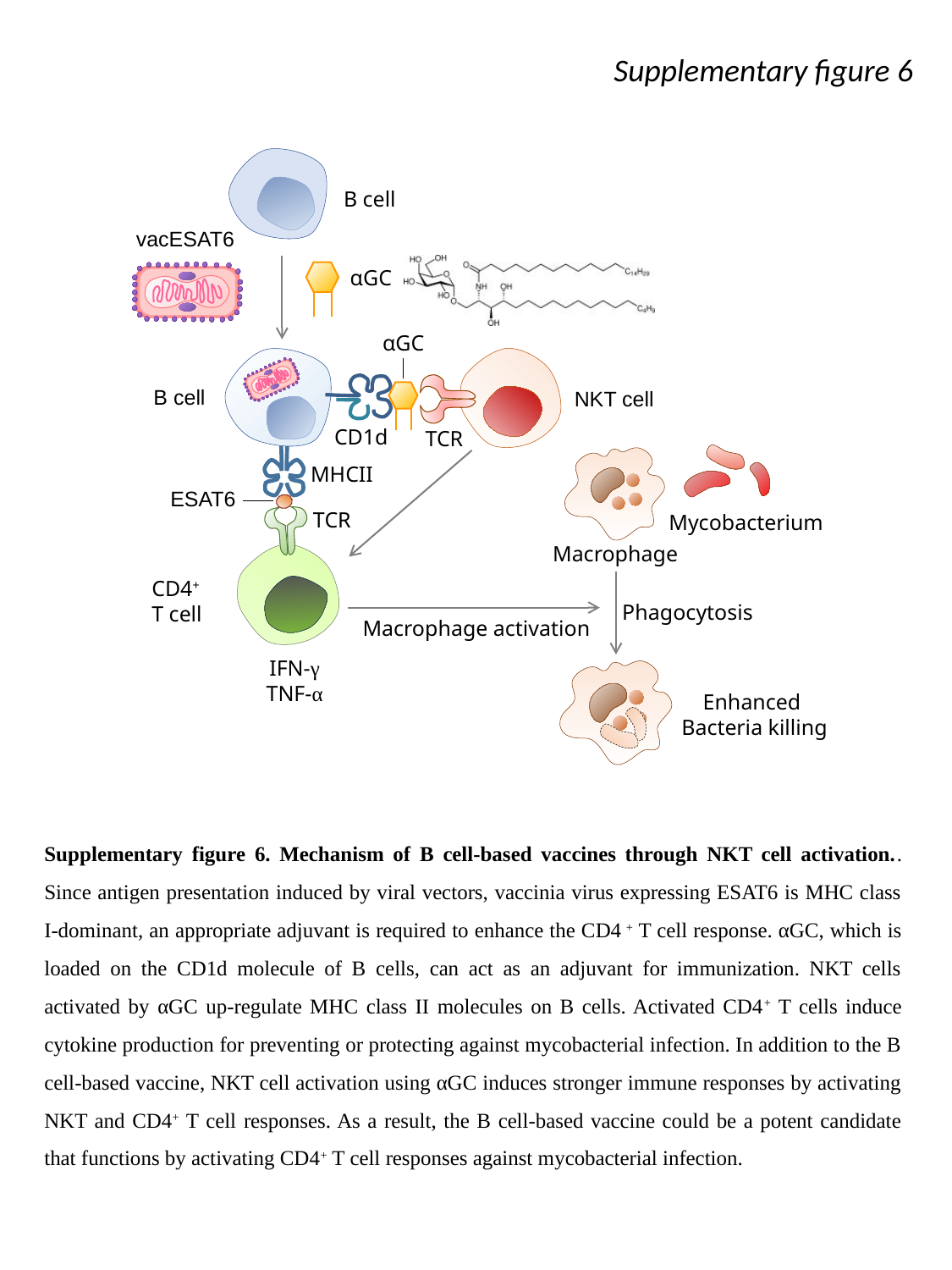

Supplementary figure 6
B cell
vacESAT6
αGC
αGC
B cell
NKT cell
CD1d
TCR
MHCII
ESAT6
TCR
Mycobacterium
Macrophage
CD4+
T cell
Phagocytosis
Macrophage activation
IFN-γ
TNF-α
Enhanced
Bacteria killing
Supplementary figure 6. Mechanism of B cell-based vaccines through NKT cell activation.. Since antigen presentation induced by viral vectors, vaccinia virus expressing ESAT6 is MHC class I-dominant, an appropriate adjuvant is required to enhance the CD4 + T cell response. αGC, which is loaded on the CD1d molecule of B cells, can act as an adjuvant for immunization. NKT cells activated by αGC up-regulate MHC class II molecules on B cells. Activated CD4+ T cells induce cytokine production for preventing or protecting against mycobacterial infection. In addition to the B cell-based vaccine, NKT cell activation using αGC induces stronger immune responses by activating NKT and CD4+ T cell responses. As a result, the B cell-based vaccine could be a potent candidate that functions by activating CD4+ T cell responses against mycobacterial infection.
